# Isolated Central Nervous System Presentation of Early T-Cell Precursor Acute Lymphoblastic Leukemia/Lymphoma: A Rare Case of Exclusive Brain Involvement

**DOI:** 10.1155/crh/7634316

**Published:** 2025-07-03

**Authors:** Violet O. Swart, Behyar Zoghi

**Affiliations:** Department of Bone Marrow Transplant, Methodist Hospital, San Antonio, Texas, USA

## Abstract

Early T-cell precursor acute lymphoblastic leukemia/lymphoma (ETP-ALL/LBL) is a high-risk and biologically distinct subset of T-cell acute lymphoblastic leukemia, typically characterized by leukemic involvement of bone marrow, peripheral blood, and mediastinal structures. Central nervous system (CNS) involvement is typically a secondary manifestation [1, 2, 3]. We report a rare case of isolated CNS presentation of ETP-ALL/LBL in a 73-year-old woman who presented with progressive neurological symptoms. Imaging revealed a large right frontal extra-axial mass. Systemic evaluation, including peripheral blood flow cytometry and CT imaging of chest, abdomen, and pelvis, showed no evidence ofsystemic disease. Surgical resection and subsequent histopathology confirmed the diagnosis of ETP-ALL/LBL. A bone marrow biopsy was deffered to the patient's preference and rapid clinical deterioration. This case underscores the diagnostic and therapeutic challenges of CNS-restricted leukemic presentations and highlights the need for early recognition and CNS-directed diagnostic evaluation.

## 1. Introduction

Early T-cell precursor acute lymphoblastic leukemia/lymphoma (ETP-ALL/LBL), first described in 2009, accounts for 10%–15% of T-ALL cases and is characterized by an immature T-cell phenotype with stem cell and myeloid marker expression. It is associated with poor prognosis, high relapse rates, and treatment resistance. ETP-ALL/LBL typically presents with systemic findings such as leukocytosis, bone marrow infiltration, hepatosplenomegaly, and mediastinal lymphadenopathy. Central nervous system (CNS) involvement, when present, usually represents a secondary site of disease progression [4, 5, 7].

This case describes an exceedingly rare presentation of ETP-ALL/LBL confined to the CNS, in the absence of systemic disease. These atyical manifestations create significant diagnostic uncertainty and challenge conventionaltreament algorithms. They also raise important questions about disease biology, optimal therapeutic approaches, and strategies for early detection.

## 2. Case Presentation

**Figure 1:**
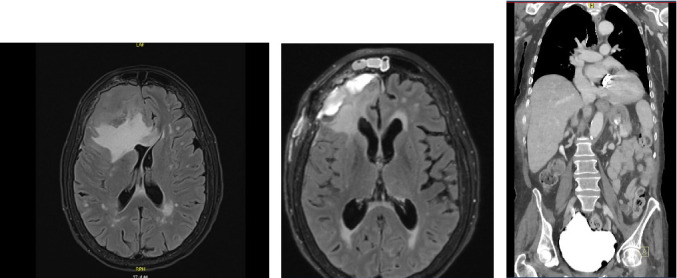
(a and b) Axial T2-FLAIR MRI showing right frontal extra-axial mass (8 × 6 × 6 cm) with midline shift. (c) CT abdomen shows no hepatosplenomegaly; the liver appears prominent, but no enlargement was measured.

A 73-year-old woman with a history of aortic valve replacement in 2008 presented with worsening headache, imbalance, weakness, and a recent fall. She denied fever, night sweat, weight loss, or other systemic symptoms. Her physical exam revealed right-sided weakness, and initial CT of the head revealed a large right frontal mass with midline shift. MRI of the brain (Figures 1(a) and 1(b)) demonstrated an 8 × 6 × 6 cm lobulated extra-axial mass in the right anterior cranial fossa, with significant surrounding edema and a 0.9 cm midline shift to the left.

Subsequent CT of the chest, abdomen, and pelvis showed no evidence of lymphadenopathy or visceral involvement (Figure 1(c)). Peripheral blood flow cytometry revealed no abnormal lymphoid population. A bone marrow biopsy was not performed, as the patient declined further invasive procedures during postoperative recovery. This decision was also influenced by the lack of systemic disease and the patient's rapid neurological deterioration aftrer surgery.

**Figure 2:**
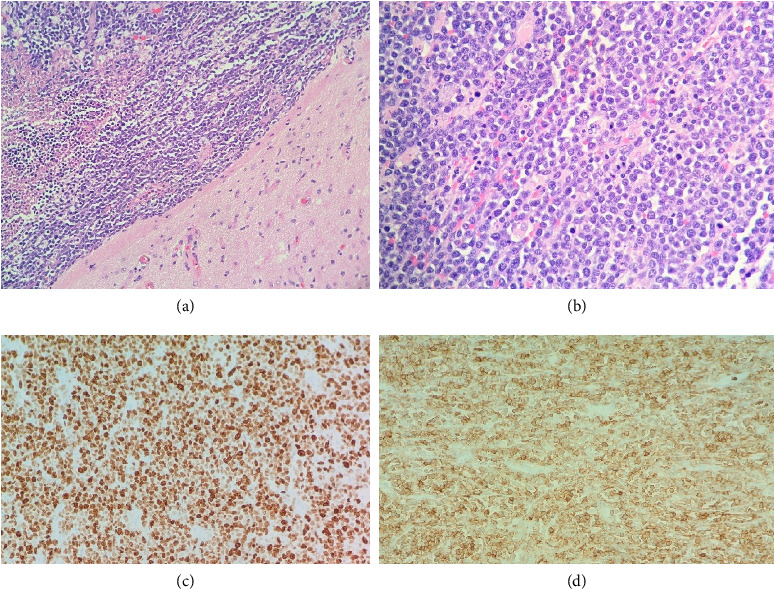
(a) H&E 40×, showing cortical brain involvement with blastoid cells and necrosis. (b) H&E 100×, showing diffuse lymphoid infiltration. (c) Ki-67 (MIB-1) IHC with > 95% proliferation. (d) CD3 and CD4 IHC showing diffuse positivity. Images provided by Dr. Alan F. Brown, department of pathology, Methodist Hospital.

The patient underwent right frontal craniotomy with gross total resection of the tumor. Histopathology revealed diffuse infiltration of the cortical brain parenchyma by medium-to-large blastoid lymphoid cells (Figures 2(a), 2(b), 2(c), and 2(d)). Immunohistochemistry demonstrated positivity for CD3, CD4, CD7, CD34 (57%), CD43, CD45Rb, BCL-2, c-MYC, and CD117. The Ki-67 proliferation index was > 95%, consistent with a diagnosis of ETP-ALL/LBL. Flow cytometry of the brain mass confirmed these findings.

Despite initial recovery, the patient developed progressive neurological deterioration, aspiration pneumonia, and sepsis. She passed away approximately 1 month after surgery.

## 3. Discussion

This case illustrates an isolated CNS presentation of ETP-ALL/LBL, a rare and diagnostically challenging manifestation of an already aggressive leukemia subtype. The absence of systemic disease, normal peripheral flow cytometry, and lack of marrow involvement complicate timely diagnosis.

Three key diagnostic lessons emerge:

- Histopathology and immunophenotyping are essential: CNS masses of lymphoid origin require early biopsy and detailed immunophenotyping.
- Peripheral blood may be misleading: Negative systemic studies do not preclude the presence of hematological malignancy, particularly in cases confined to the CNS.
- Early CNS-directed evaluation is crucial: In patients with undifferentiated brain masses, CSF analysis, advanced imaging, and flow cytometry should be considered, especially in older patients.

The pathophysiology of CNS-restricted leukemic presentations remains poorly understood but may involve selective migration of leukemic precursors across the blood–brain barrier via adhesion molecules and chemokine interactions (e.g., CXCR4, VLA-4, and ICAM-1). Mutations in NOTCH1, FLT3, DNMT3A, and PHF6 may also play a role in CNS homing and immune evasion [6, 8, 10].

No systemic leukemia-directed therapy was administered in this case due to the delayed diagnosis and clinical deterioration. Standard ETP-ALL/LBL regimens typically involve high-dose chemotherapy and stem cell transplant, often with CNS prophylaxis. However, in isolated CNS cases, the role of CNS-penetrant agents such as intrathecal methotrexate, high-dose cytarabine, and venetoclax-based regimens remains to be defined. Emerging investigational strategies, including CAR T-cell therapy targeting CD7 and CD34, and small molecule inhibitors of JAK/STAT and FLT3 pathways, may hold promise in molecularly defined subgroups [9].

## 4. Conclusion

This case represents one of the very few documented instances of ETP-ALL/LBL presenting exclusively in the CNS. It highlights the importance of early diagnostic suspicion, thorough CNS-directed evaluation, and personalized therapeutic strategies. Further research is needed to define the biology of CNS-restricted leukemia and identify effective CNS-penetrant therapies.

## Data Availability

The data that support the findings of this study are available from the corresponding author upon reasonable request.
